# CD36 Deficiency Leads to Choroidal Involution via COX2 Down-Regulation in Rodents

**DOI:** 10.1371/journal.pmed.0050039

**Published:** 2008-02-19

**Authors:** Marianne Houssier, William Raoul, Sophie Lavalette, Nicole Keller, Xavier Guillonneau, Barbara Baragatti, Laurent Jonet, Jean-Claude Jeanny, Francine Behar-Cohen, Flavio Coceani, Daniel Scherman, Pierre Lachapelle, Huy Ong, Sylvain Chemtob, Florian Sennlaub

**Affiliations:** 1 Institut National de la Santé et de la Recherche Médicale U872, Paris, France; 2 Centre de Recherche des Cordeliers, Université Pierre et Marie Curie, UMR S 872, Paris, France; 3 Université Paris Descartes, UMR S 872, Paris, France; 4 Institut National de la Santé et de la Recherche Médicale U592, Paris, France; 5 Université Pierre et Marie Curie, UMR S 592, Paris, France; 6 Scuola Superiore Sant'Anna and Institute of Clinical Physiology CNR, Pisa, Italy; 7 Institut National de la Santé et de la Recherche Médicale U640/UMR 8151, Centre National de la Recherche Scientifique, Université René Descartes, Paris, France; 8 Department of Pediatrics, Ophthalmology and Pharmacology, Research Center, Hôpital Ste Justine, Montréal, Québec, Canada; 9 Faculty of Pharmacy, Université de Montréal, Montréal, Québec, Canada; Jules Gonin Eye Hospital, Switzerland

## Abstract

**Background:**

In the Western world, a major cause of blindness is age-related macular degeneration (AMD). Recent research in angiogenesis has furthered the understanding of choroidal neovascularization, which occurs in the “wet” form of AMD. In contrast, very little is known about the mechanisms of the predominant, “dry” form of AMD, which is characterized by retinal atrophy and choroidal involution. The aim of this study is to elucidate the possible implication of the scavenger receptor CD36 in retinal degeneration and choroidal involution, the cardinal features of the dry form of AMD.

**Methods and Findings:**

We here show that deficiency of CD36, which participates in outer segment (OS) phagocytosis by the retinal pigment epithelium (RPE) in vitro, leads to significant progressive age-related photoreceptor degeneration evaluated histologically at different ages in two rodent models of CD36 invalidation in vivo (Spontaneous hypertensive rats (SHR) and CD36^−/−^ mice). Furthermore, these animals developed significant age related choroidal involution reflected in a 100%–300% increase in the avascular area of the choriocapillaries measured on vascular corrosion casts of aged animals. We also show that proangiogenic COX2 expression in RPE is stimulated by CD36 activating antibody and that CD36-deficient RPE cells from SHR rats fail to induce COX2 and subsequent vascular endothelial growth factor (VEGF) expression upon OS or antibody stimulation in vitro. CD36^−/−^ mice express reduced levels of COX2 and VEGF in vivo, and COX2^−/−^ mice develop progressive choroidal degeneration similar to what is seen in CD36 deficiency.

**Conclusions:**

CD36 deficiency leads to choroidal involution via COX2 down-regulation in the RPE. These results show a novel molecular mechanism of choroidal degeneration, a key feature of dry AMD. These findings unveil a pathogenic process, to our knowledge previously undescribed, with important implications for the development of new therapies.

## Abstract 2

Florian Sennelaub and colleagues show that CD36 deficiency leads to choroidal involution, a key feature of "dry" age-related macular degeneration, via COX-2 down-regulation in the retinal pigment epithelium.

## Editors' Summary

**Background.:**

Age-related macular degeneration (AMD) is the leading cause of blindness in the elderly in industrialized countries. The macula is the central region of the retina, the tissue at the back of the eye that detects light and converts it into electrical messages that are sent to the brain. In the commonest form of AMD—“dry” AMD—the light-sensitive cells in the retina (the photoreceptors) gradually die. This degeneration might occur because of damage to the retinal pigment epithelium (RPE). This layer of dark cells lies between the photoreceptors and the choroid, the layer of the eye that contains blood vessels and brings oxygen to the retina. The RPE keeps the retina healthy by transferring the right amount of oxygen and nutrients from the choroid to the retina and by removing worn-out photoreceptor outer segments (the part of the photoreceptor that actually absorbs light) in a process called phagocytosis (engulfment and digestion). In addition to photoreceptor degeneration and RPE shrinkage, a layer of the choroid rich in small blood vessels (the choriocapillaris) also shrinks in dry AMD. For affected individuals, all these changes (which experts describe as retinal atrophy and choroidal involution) mean that the sharp central vision that is needed for reading and driving is destroyed, leaving only dim, burred images or a black hole at the center of the vision.

**Why Was This Study Done?:**

Little is known about the molecular mechanisms that underlie dry AMD and, consequently, there is no cure for it. In this study, the researchers have tested whether a molecule called CD36, which is expressed on the surface of RPE cells, is involved in dry AMD. CD36 is a scavenger receptor—which means it binds many potentially harmful molecules including oxidized fats (which are present in the photoreceptor outer segments) and is involved in their phagocytosis. Phagocytosis itself induces the expression of several proteins in the RPE cells, including COX2, a “proangiogenic” protein that stimulates the growth of blood vessels. Putting this information together, the researchers hypothesized that a defect in CD36 might cause the characteristic retinal atrophy (by preventing the phagocytosis of worn-out photoreceptor outer segments) and choroidal involution (by preventing the induction of COX2 expression and consequently the maintenance of the blood vessels in the choroid) of dry AMD.

**What Did the Researchers Do and Find?:**

The researchers first show that retinal degeneration occurs in rats and mice that express no CD36. This degeneration (which included a reduction in the thickness of the retina, the presence of irregularly shaped photoreceptor outer segments, and the detachment of these structures from the RPE) was seen in old but not young animals. Choroidal involution was also seen in these CD36-deficient animals. This change was present in young mice and rats but increased with age so that by one year old, the choriocapillaris looked moth-eaten. Next, the researchers show that although RPE cells taken from normal animals and grown in dishes were able to make COX2 in response to exposure to purified photoreceptor outer segments, RPE cells from CD36-deficient animals did not. The expression of vascular endothelial growth factor (VEGF; a protein that is needed for normal choroidal development and whose expression is controlled by COX2) showed a similar pattern. Finally, the researchers report that COX2 deficiency in mice caused similar age-dependent choroidal involution and similar effects on VEGF expression in RPE cells as CD36 deficiency.

**What Do These Findings Mean?:**

These findings show that CD36 deficiency leads to progressive, age-related degeneration of photoreceptors and choroidal involution in rats and mice. They also show that CD36 deficiency causes this choroidal involution, the key feature of dry AMD, because it leads to down-regulation of COX2 expression (and subsequently reduced VEGF expression) in the RPE. Researchers now need to find out whether this mechanism for the development of dry AMD holds in people—what happens in animals does not necessarily happen in people. If it does, pharmacological activation of CD36 or restoration of CD36 expression in the RPE might eventually provide a way to treat dry AMD.

**Additional Information.:**

Please access these Web sites via the online version of this summary at http://dx.doi.org/10.1371/journal.pmed.0050039.

- MedlinePlus provides links to information on macular degeneration and an encyclopedia page on macular degeneration (in English and Spanish)
- Pages on the US National Institutes of Health NIH SeniorHealth site provides text and spoken information about AMD
- The US National Eye Institute and the UK Royal National Institute of Blind People also provide information about AMD
- Wikipedia has pages on the retina, photoreceptor cells, retinal pigment epithelium, and choroid (note that Wikipedia is a free online encyclopedia that anyone can edit; available in several languages)

## Introduction

Age-related macular degeneration (AMD) is the leading cause of vision loss among older adults in industrialized countries [1]. The most prominent pathologic features of AMD involve lesions of the photoreceptors, retinal pigment epithelium (RPE), Bruch membrane, and the choriocapillaris [2]. Early AMD is characterized by drusen (focal deposits in Bruchs membrane [BM]) and basal deposits (diffuse sub-RPE debris in BM) and changes in RPE pigmentation. There are two clinical forms of late AMD: the “wet” form, defined by choroidal neovascularization, and the “dry” form, characterized by circumscribed atrophy of RPE and thinning and obliteration of the choriocapillary layer (geographic atrophy) [2,3]. Although most of the cases of legal blindness in AMD [4] are a consequence of choroidal neovascularization (the wet form), the vast majority of patients first develop severe visual impairment secondary to geographic atrophy seen in the dry form. Current research and emerging therapies (anti-vascular endothelial growth factor [VEGF] treatments) mainly focus on the neovascular aspect of wet AMD, and little treatment is available to patients with the atrophic, dry form. The basic mechanisms underlying AMD, and particularly geographic atrophy and choroidal involution, remain elusive.

Physiologically, the RPE cells transfer oxygen and nutrients from the choroidal circulation to the outer retina (external hemato-retinal barrier). They engulf, degrade, and recycle used photoreceptor outer segments (OS), and clear the debris to the choroidal circulation. Phagocytosis of spent OS is critical for the long-term maintenance of the retina [5,6] and is dependent on a tyrosine kinase receptor (i.e., for c-mer proto-oncogene tyrosine kinase [MERTK]) [7,8] and integrins [9]. CD36 is a scavenger receptor [10] that is expressed in RPE cells [11], among others. It is involved in phagocytosis [12] particularly of oxidized lipids [13]. Phagocytosis in turn “induces” a number of genes expressed in RPE [14] such as the proangiogenic *cyclooxygenase 2 COX2* [15] (also known as *prostaglandin-endoperoxide synthase 2 [PTGS2]*), which controls *VEGF* expression in various cells [16]. In addition, the multiple-ligand receptor CD36 is the main antiangiogenic receptor of thrombospondin-1 (TSP-1) [17].

Collectively, CD36 dysfunction in vivo could participate in retinal degeneration, alter the expression of essential proangiogenic factors in the RPE, or lead to neovascularization as a result of the lack of TSP-1 signaling in vascular endothelium. Spontaneous hypertensive rats (SHRs) develop visual dysfunction and retinal degeneration independent of hypertension [18,19] as well as choroidal involution [27]. These changes could be secondary to invalidating *CD36* mutations [20] found in (certain) SHR strains. To decipher the role of CD36 in chorioretinal homeostasis we analyzed eyes from SHR strains bearing the invalidating *CD36* mutations and from normotensive CD36^−/−^ mice.

## Materials and Methods

### Animals

CD36^−/−^ mice [21] and COX2^−/−^ mice [22] and their wild-type controls were housed at local animal facilities under 12 h light–12 h dark cycles and fed ad libitum. CD36^−/−^ mice and COX2^−/−^ mice were back-crossed on a C57Bl6 background for eight generations. CD36^−/−^ mice and their controls were reproduced separately thereafter. COX2^−/−^ and COX2^+/+^ mice were genotyped littermates from heterozygote genitors. Spontaneous hypertensive rats (SHRs) and Wistar controls were purchased from the Janvier breeding center (Le Genest-St-Isle, France). Animal experiments were approved by the Institutional Animal Care and Use Committee of the University Paris V, Paris, France.

### Western Blots

10-d-old SHRs (*n* = 6) and Wistar rats (*n* = 6) were humanely killed and eyes enucleated. The eyes were dissected and RPE/choroid/sclera complexes were sonicated in ice-cold lysis buffer (Tris-HCl 50 mM [pH 6.8], 2% SDS, and 2 mM PMSF as antiprotease; the RPE is firmly attached to the choroid in the dissecting process). Protein preparation, electrophoresis, and transfer to nitrocellulose membrane were performed as previously described [23]. Primary antibodies used were mouse monoclonal CD36 FA6–152 (1:500; Abcam) and with monoclonal anti-β-actin (1:5000, Santa Cruz) to control for protein loading. proteins were revealed by corresponding secondary horseradish peroxidase-conjugated antibodies.

### Immunohistochemistry

Eyes were fixed in paraformaldehyde 4% in PBS for 15 min at room temperature (RT) and rinsed in PBS before embedded in OCT (Tissue Tek). Frozen transverse sections 10 μm thick were cut and permeabilized for 10 min in 1% Triton X-100. Postfixation was performed with methanol or ethanol, depending on the antibody used. Immunolabeling with primary antibodies (1:100) rabbit polyclonal CD36 (Santa Cruz), rabbit polyclonal VEGF (Santa Cruz), rabbit polyclonal COX2 (Biomol), and endothelial cell marker Bandeiraea simplicifolia agglutinin 1 [24] (BSA-1, Sigma) was performed overnight at RT. After washing in PBS, secondary antibodies coupled with Alexa Fluor 488 (1:100, Molecular Probes) were applied for 2 h at RT. Nuclei were labeled with DAPI (1:4000, Sigma-Aldrich) and sections were mounted with Gelmount (Biomeda). Fluorescence was observed with an Olympus BX51 microscope and photographs were taken using the same exposure times and contrast settings or a confocal microscope (Zeiss LSM 510 Laser scanning). All immunostainings were repeated at least three times, and staining without primary antibody served as negative controls.

### Histology and Electron Microscopy

#### Electron microscopy.

Eyes were fixed for 1 h in 2.5% glutaraldehyde in cacodylate buffer (0.1 M, pH 7.4). After 1 h, the eyeballs were dissected, fixed for another 3 h, postfixed in 1% osmium tetroxide in cacodylate buffer, and dehydrated in graduated ethanol solutions. The samples were included in epoxy resin and oriented. Semi-thin sections (1 μm), obtained with an ultramicrotome (Reichert Ultracut E [Leica]), were stained by toluidine blue, examined with a light microscope, and measurements photoreceptor layer thickness were made. Ultra-thin sections (80 nm) were contrasted by uranyl acetate and lead citrate and were observed with an electron microscope JEOL 100 CX II (JEOL) with 80 kV.

#### Paraffin sections.

The eyes were enucleated, fixed in Bouin's fixative for 24 h, and embedded in paraffin. Sagittal sections (7 μm) were cut in parallel to the optic nerve and stained with periodic acid Schiff (PAS) and hemalun. Photoreceptor layer thickness was measured on four sections containing the optic nerve 14 μm apart from one another. using digitalized images and Image J Software. The data were averaged for each eye, and the mean values from the individual eyes were statistically analyzed. Investigators performed measurements unaware of the provenance of the samples.

### Reverse Transcription and Real-Time Polymerase Chain Reaction

Total RNA was isolated with RNeasy Mini Kit (Qiagen). Single-stranded cDNA was synthesized from total RNA (pretreated with DNaseI amplification grade) using oligo-dT as primer and superscript reverse transcriptase (Invitrogen). Subsequent real-time polymerase chain reaction (RT-PCR) was performed using cDNA, qPCR SuperMix-UDG Platinum SYBR Green (Invitrogen), and the following primers (0.5 pmol/μl): Actin sense, 5′-AAA GAA AGG GTG TAA AAC GCA G-3′; actin antisense, 5′-AAA GAC CTC TAT GCC AAC ACA G-3′; CD36 sense: 5′-GAC AAT CAA AAG GGA AGT TG-3′; CD36 antisense: 5′-CCT CTC TGT TTA ACC TTG AT-3′; VEGF sense: 5′-TGG GAT GGT CCT TGC CTC-3′; VEGF antisense: 5′-TCG CTG GAG TAC ACG GTG GT-3′; COX2 sense: 5′-TGC TAC CAT CTG GCT TCG GGA G-3′; COX2 antisense: 5′-ACC CCT CAG GTG TTG CAC GT-3′.

PCR reactions were performed in 40 cycles of 15 s at 95 °C, 45 s at 60 °C. Product was not generated in control reactions in which reverse transcriptase was omitted during cDNA synthesis.

### RPE Primary Culture

Ten day-old pups (Wistar rat and SHR) were humanely killed and eyes dissected and enucleated. Eyes were maintained at room temperature overnight in Dulbecco's Modified Eagle's Medium (DMEM, Invitrogen) then incubated 45 min with 2 mg/ml trypsin/collagenase I at 37 °C. After trypsin inhibition with DMEM containing 10% fetal calf serum (FCS), the RPE layer was harvested. The RPE was plated in 12-well plates at a rate of RPE from one eye per well in DMEM containing 10% FCS, 1% penicillin/streptomycin, and 0.2% fungizone. Cells were maintained for 12 d before the phagocytosis assay.

### OS Isolation and Phagocytosis Assay and CD36 Activation

OS were isolated following established protocols [25]. Briefly, 20 pig's retinas were dissected and homogenized in 20% sucrose buffer, 20 mM Tris, 2 mM MgCl_2_, 0.13 mM NaCl (pH 7.2). Retina samples were centrifuged on a sucrose gradient (50%, 27%) 1 h at 38,000 rpm, and OS were harvested at the ring interface and diluted in DMEM. Centrifugation 10 min at 8,000 rpm was performed and the pellet was resuspended in DMEM to obtain a stock solution of 10^8^ OS/ml.

Confluent RPE monolayers were challenged with 150 μl of 10^8^ OS/ml. After 1h 850 μl of complete medium was added. Cells were washed and 350 μl of RLT lysis buffer (RNeasy mini kit, Qiagen) was added for RNA extraction at 0 and 6 h.

The CD36 antibody FA6–152 antibody (Abcam) can activate CD36 by dimerization of the receptor as previously shown [12]. CD36 was activated with 20 μg/ml of FA6–152 antibody as described [12]. RPE cells from Wistar rats or SHRs were incubated with either FA6–152 or control antibody in DMEM for 4 h and mRNA was prepared as described above.

COX2 inhibition was achieved by a 30 min preincubation with 10^−^6 M DUP697; this concentration was maintained throughout the experiments.

### Vascular Corrosion Casts

Animals were killed by CO_2_ inhalation followed by a thoracotomy. Venous catheter was introduced into the aorta through the left heart ventricle, and the right auricle was cut to allow evacuation of injected products. A perfusion was performed with a mixture of red Mercox resin and catalyst (Ladd Research). Eyes were extracted and lenses were removed. Tissues were conserved overnight at 37 °C in PBS to allow complete polymerization, and then digested by 5% KOH for 2 wk at 37 °C until only the vascular corrosion casts remained. Distilled water was used to remove salt and the mold was dried. Only corrosion casts with completely filled iris vessels were used, to exclude corrosion casts from incomplete perfusion. Retinal vasculature was removed using forceps. The specimens were mounted on SEM stubs, coated with gold palladium, and scanned at an accelerating voltage of 117 kV. In order to measure the thickness of the choriocapillary lumen, corrosion casts were cut paracentrally (1 mm from the aperture of the optic nerve) and positioned for perpendicular views of the choriocapillaries. To analyze the intercapillary space (avascular area), the casts were positioned for frontal views of the choriocapillaries. Electron micrographs were scanned and analyzed using Image J Software (http://rsb.info.nih.gov/ij/). The avascular area was measured on frontal views and expressed as the percentage of intercapillary surface (space between the plastic capillary casts) of the whole area. Thickness of choriocapillaries was measured on perpendicular views of the cast from the retinal to scleral side of the choriocapillary cast.

### Statistical Analysis

Variance was analyzed by Kruskal-Wallis test. Data between two groups were compared with nonparametric Mann Whitney U-test. All analysis and graphic representation were performed with Prism software (version 4.0c; GraphPad Software), and values are represented as mean ± standard error of the mean (SEM). P values were calculated for a confidence interval of 95% and *P* values of less than 0.05 were considered significant.

## Results

### Retinal Degeneration in CD36-Deficient Animals

In vitro participation of CD36 in the phagocytosis of OS by human [11] and rat RPE cells in vitro [12] has previously been described. However, the involvement of CD36 in phagocytosis in vivo has been unknown. To assess the role of CD36 in phagocytosis and retinal homeostasis in vivo, we examined two animal models: an albino SHR strain containing several CD36 mutations that lead to undetectable levels of CD36 expression in several tissues [20], and pigmented normotensive CD36^−/−^ mice [26].

**Figure 1:**
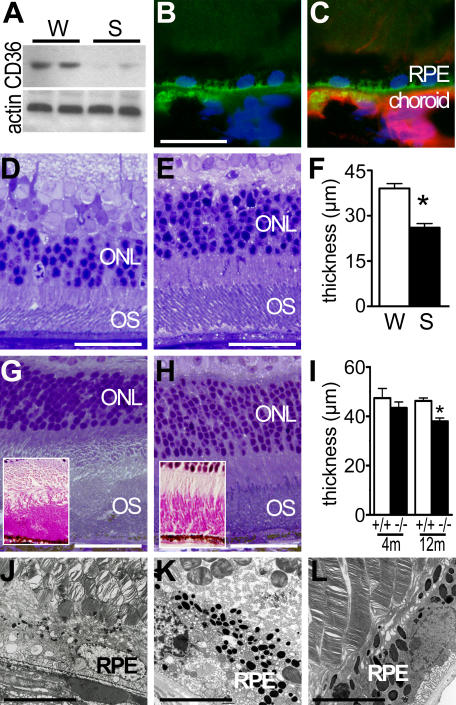
Retinal Degeneration in CD36-Deficient Animals (A) CD36 Western blot analysis of RPE/choroids complexes from Wistar rats (W) and SHRs S) (*n* = 4 eyes per group). (B and C) CD36 expression (green fluorescence) (B) and double-labeling with vascular marker BSA-1 (CD36 [green], BSA-1 [red], DAPI [blue]) (C) in CD36^+/+^ mice (representative picture of three independent experiments). (D and E) Hemalun stained semi thin sections of 10-mo-old CD36-deficient SHRs (D) and control Wistar (W) rats (E). (F) Outer nuclear layer measurements of 10-mo-old Wistar rats (W; *n* = 6) SHRs (S; *n* = 8) (*p < 0.0001). (G and H) Hemalun-stained semi-thin sections (and periodic acid Schiff-stained paraffin sections [inset]) of 1-y-old CD36^−/−^ mice (G) and age-matched WT mice (H). (I) ONL thickness measurements in eyes of CD36^−/−^ (black bars; *n* = 10) and CD36^+/+^ (white columns; *n* = 6) mice at different ages (*p = 0.0095 significant difference at 12 mo). (J–L) Transmission electron microscopy of the RPE and outer segments in SHRs (J), and CD36^−/−^ mice (K) and a CD36-expressing congener strain (CD36^+/+^ mice) (L). Results are representative of at least three independent experiments. Scale bar: B, C, D, E, G, and H = 50 μm; J–L = 5μm.

Western blot analysis of RPE/choroid complexes showed greatly diminished CD36 protein expression in the eyes of SHRs compared to the Wistar rat control strain in vivo (Figure 1A). CD36 localization in mice was analyzed using specific antibodies. CD36 was expressed in the basal aspect of the RPE (Figure 1B) and in choroidal vessels (CD36 [green] and BSA-1 [red] double stain).

Semi-thin sections of 10-mo-old SHRs (Figure 1D) revealed an irregular outer nuclear layer (ONL) and patchy retinal degeneration. OS morphology was disrupted compared to the regularly shaped photoreceptors of control animals (Figure 1E). Quantification of the ONL thickness revealed a significant 26% reduction in ONL at this age (Figure 1F). Similarly, semi-thin sections of 12-mo-old CD36^−/−^ mice (Figure 1G) showed a thinned irregular outer nuclear layer (ONL) and ill-defined limit between inner and outer photoreceptor segments; OS were irregular and accumulated in some areas as seen in periodic acid Schiff stains (Figure 1G inset). Whereas 12-mo-old CD36^+/+^ mice showed regularly shaped photoreceptors (Figure 1H). Although regional differences in degenerative changes were observed, overall ONL was 17% thinner (*p* < 0.05) at 12 mo of age; this was not yet observed at 4 mo of age (Figure 1I).

Detailed morphological evaluation by electron microscopy (EM) of 10-mo-old SHRs (Figure 1J) and 12-mo-old CD36^−/−^ mice (Figure 1K) showed OS detachment from the RPE villi. Strikingly, OS appeared in oblique as well as in perpendicular planes of sagittal sections of the eyes (note Figure 1J and 1K sagittal RPE choroids section plane). In control animals (Figure 1L, 12-mo-old CD36^+/+^ mouse), OS were in tight contact with RPE villi and were longitudinal or slightly oblique throughout the sagittal eye sections; perpendicularly (cross)-sectioned OS were never observed in control animals. These differences might be due to OS overgrowth in CD36 deficiency secondary to RPE phagocytosis deficiency. The elongated OS in these animals could explain the accumulation of periodic acid Schiff material (Figure 1G inset) and the disorientation of the OS (on EM; Figure 1J and 1K).

### Choroidal Involution in CD36-Deficient Animals

Choroidal involution is a main feature of dry AMD [2]. Little is known of the molecular mechanisms leading to choroidal involution. Interestingly, choroidal involution was reported in SHRs several decades ago [27], prior to knowledge of their CD36 status. To study the influence of CD36 deficiency on choroidal integrity we analyzed choroids of CD36-deficient SHRs and CD36^−/−^ mice.

**Figure 2:**
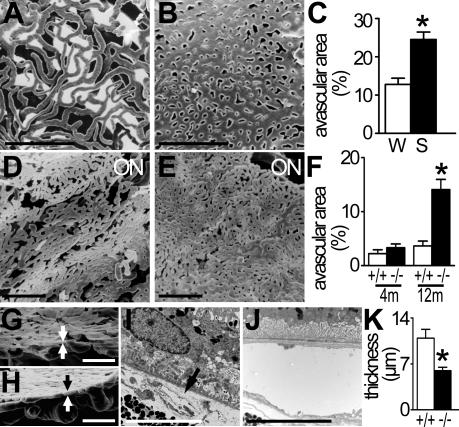
Choroidal Degeneration in CD36-Deficient Animals (A and B) Micrographs of the retinal aspect of choriocapillaries in a frontal view of corrosion casts by scanning electron microscopy. Choroidal vessels (darker grey) can be seen through the intercapillary spaces of the choriocapillaries in 4-mo-old SHRs (A) but not in age-matched Wistar rats (B). (C) Quantification of intercapillary space expressed as avascular area in 4-mo-old Wistar rats (W) and SHRs (S) (*n* = 5 eyes/group; *p = 0.0027). (D and E) Frontal view of the retinal aspect of choriocapillaries of 12-mo-old CD36^−/−^ (D) and CD36^+/+^ (E) mice show defects in the capillary bed of CD36^−/−^ mice. (F) Quantification of the avascular area over time (ages 4 mo versus 12 mo) of CD36^+/+^ (*n* = 6) and CD36^−/−^ (*n* = 8) mouse eyes; *p = 0.0286 significant difference at 12 mo). (G and H) Perpendicular view of choriocapillaries (indicated between arrows) and large choroidal vessels on cross-sectional cuts of pericentral area of CD36^−/−^ (G) and CD36^+/+^ control (H) mice. (I and J) Transmission electron microscopy of choriocapillaries of CD36^−/−^ (arrow) (I) and CD36^+/+^ (J) mice. (K) Quantification of capillary thickness of 12-mo-old CD36^−/−^ (*n* = 10) and CD36^+/+^ (*n* = 8) mouse eyes (*p = 0.0062). Results are representative of at least three independent experiments. +/+, wild-type animals, white columns; −/−, CD36-deficient animals, black columns; m, month; ON, optic nerve. Scale bar: A, B, D, E, G, and H = 100 μm; I and J = 5 μm.

Vascular corrosion casts of the choriocapillaries of 4-mo-old SHRs revealed a vascular rarefaction of the choriocapillaries (Figure 2A) compared to age matched control Wistar rats (Figure 2B). Correspondingly, quantification of intercapillary space revealed a significant increase in avascular area in SHRs (Figure 2C). Similarly, choriocapillaries of 12-mo-old CD36^−/−^ mice showed capillary dropout and a moth-eaten appearance (Figure 2D) compared to the dense microvasculature of CD36^+/+^ mice (Figure 2E); this was reflected by an increase in avascular area of choroids of 12-mo-old mice CD36^−/−^ mice compared to CD36^+/+^ mice; a tendency toward an increase in avascularity was already detected by 4 mo of age, although this was not yet statistically significant (Figure 2F). In addition, cross-sectional views of the vascular corrosion casts showed severe thinning of choroids from CD36^−/−^ (Figure 2G) compared to control mice (Figure 2H). This involution was also observed using transmission electron microscopy, where choriocapillaries of CD36^−/−^ mice were either missing or exhibited severely diminished thickness compared to those of CD36^+/+^ mice (Figure 2I–2K). In contrast, capillary density of other organs such as the skin, brain, and ocular muscles were not affected (unpublished data).

### OS-Induced COX2 and VEGF Expression in RPE is CD36-Dependent

In vivo RPE cells express prosurvival/proangiogenic factors such as COX2 that may be necessary for choriocapillary integrity [23] upon OS stimulation [15]. The COX2 expression in the RPE appears to be phagocytosis dependent, as RPE primary cell cultures express COX2 once stimulated with OS [15]. As CD36 influences phagocytic activity in vitro [12] we surmised that CD36 expression influences that of COX2 in RPE.

**Figure 3:**
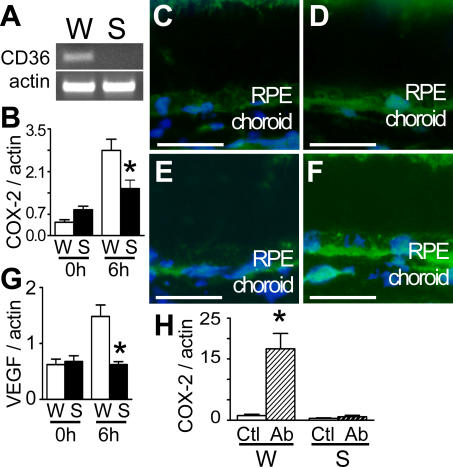
OS-Induced COX2 and VEGF Expression in RPE is CD36 Dependent (A–G) RT-PCR of cDNA from primary RPE cultures from Wistar rats and SHRs (A). Relative COX2 (B) and VEGF (G) mRNA expression (measured by real time RT-PCR. *n* = 6 wells per group; (B) *p = 0.0152 significant difference between control and CD36-deficient rats at 6 h; (G) *p = 0.0087 significant difference at 6 h) in RPE cells of Wistar (W) and SHR (S) rats exposed in culture to rod outer segments. COX2 (C and D) and VEGF (E and F) immunoreactivity (green) in 4-mo-old CD36^−/−^ (C and E) and CD36^+/+^ (D and F) mice; tissues were counterstained with DAPI (nuclear stain). (H) Activation of CD36 with stimulating antibody evoked COX2 expression on RPE cell cultures from Wistar rats (W) and SHRs (measured by real time RT-PCR; *n* = 6 wells per group; *p = 0.0012 COX2 expression significantly different between control [Ctl] and antibody-treated [Ab] Wistar RPE culture). Photographs of immunohistochemical signal were taken with identical parameters in CD36^−/−^ and CD36^+/+^ mice. Results are representative of at least three independent experiments. Ab, CD36 antibody FA6–152; CTL, control; RPE, retinal pigment epithelium. Scale bar: 50 μm

Absence of CD36 mRNA in the SHR strain was verified by RT-PCR on primary RPE cells (Figure 3A). Primary RPE cell cultures from CD36-deficient SHRs and control Wistar rats were exposed to OS for different durations, and COX2 mRNA analyzed (by real-time RT-PCR). Control RPE cells exhibited significant increases in COX2 mRNA expression 6 h after stimulation (Figure 3B), as previously described [15]. In contrast, rat primary RPE cells deficient in CD36 (Figure 1A) failed to respond to OS exposure (Figure 3B). Analogously, in eyes of CD36^−/−^ mice COX2 immunoreactivity was reduced in the RPE (Figure 3C) compared to that of CD36^+/+^ mice (Figure 3D). Moreover, direct activation of CD36 even in the absence of OS, using a CD36 antibody at stimulating concentrations [12], greatly induced COX2 in RPE from CD36-expressing Wistar rats, but not in RPE from the CD36-deficient SHR strain (Figure 3H).

Because COX2 activity can control VEGF expression [16] and VEGF expression in RPE is essential for normal choroidal development [28] and possibly its homeostasis in rodents, we investigated if CD36 expression also affected that of VEGF. VEGF mRNA expression in RPE was also positively regulated by phagocytosis and blunted by CD36 deficiency (Figure 3G); likewise, RPE of CD36^−/−^ mice expressed diminished VEGF immunoreactivity (Figure 3E) compared to CD36^+/+^ mice in vivo (Figure 3F).

### COX2^−/−^ Mice Develop Choroidal Involution

**Figure 4:**
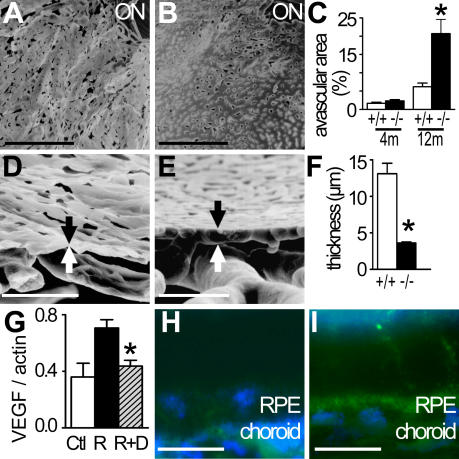
Choroidal Involution in COX2^−/−^ Mice (A and B) Micrographs of the retinal aspect of choriocapillaries in a frontal view of corrosion casts by scanning electron microscopy of 12-mo-old COX2^−/−^ (A) and COX2^+/+^ (B) mice. (C–E) Quantification of the avascular area (*n* = 6 COX2^+/+^ and *n* = 8 COX2^−/−^ eyes; *p = 0.007 COX2^−/−^ significantly different from COX2^+/+^ at 12 mo) (C). Cross-sectional cuts of pericentral choroidal corrosion casts of COX2^−/−^ (D) and COX2^+/+^ (E) mice. (F) Quantification of capillary thickness of 12-mo-old COX2^+/+^ and COX2^−/−^ mice (*n* = 6 COX2^+/+^ and *n* = 8 COX2^−/−^ eyes; *p = 0.0007). (G–I) Relative VEGF mRNA expression (by real time RT-PCR; *n* = 8 wells per group; *p = 0.0029 rod outer segments with DUP697 [R+D] significantly different from rod outer segments alone [R]) in primary RPE culture of Wistar rats (Ctl, white column), exposed to rod outer segments in absence (R, black column) or presence of the COX2 inhibitor DUP697 (10^−6^ M) (R+D, hatched column) (G). VEGF expression in 4-mo-old COX2^−/−^ (H) and COX2^+/+^ (I) mice. Photomicrographs of immunohistochemical signal were taken with identical parameters. Results are representative of at least three independent experiments. m, months; RPE, retinal pigment epithelium. Scale bar: A, B, D, and E = 100 μm; H and I = 100 μm.

On the basis of observations presented above, we surmised that COX2 expression in RPE also affected choroidal homeostasis. We proceeded to study choroidal morphology in COX2^−/−^ mice and their wild-type congeners. Compared to COX2^+/+^ mice (Figure 4B), vascular corrosion casts of 12-mo-old COX2^−/−^ mice (Figure 4A) were extremely brittle and showed a moth-eaten appearance secondary to capillary dropout as detected by the increased avascular area; these changes were not yet perceptible in 4-mo-old mice (Figure 4C). Cross-sectional views revealed severe involution of the choriocapillaris in COX2^−/−^ (Figure 4D) relative to wild-type mice (Figure 4E), as noted by the significant reduction in capillary thickness in COX2^−/−^ mice at 12 mo of age (Figure 4F). As seen in CD36^−/−^ mice, capillary morphometry in other organs (skin, brain, and ocular muscles) were not altered (unpublished data). Interestingly, in contrast to CD36^−/−^ mice, COX2^−/−^ mice did not develop degeneration of photoreceptors by 12 mo of age (ONL thickness COX2^+/+^ ± SEM [*n* = 4] = 101 ± 5.6 μm; COX2^−/−^ [*n* = 4] = 109 ± 9.8 μm)

As mentioned above, COX2 activity can regulate VEGF expression in various cells [16]. We therefore investigated if COX2 activity can influence VEGF expression in OS-exposed primary rat RPE cultures. Indeed, selective COX2 inhibition by DUP697 [29] prevented OS-induced VEGF mRNA expression (Figure 4G), but not basal VEGF expression. Similar observations were made in vivo, whereby VEGF expression in RPE was substantially reduced in COX2^−/−^ (Figure 4H) compared to COX2^+/+^ mice (Figure 4I). Altogether, the consequences of COX2 deficiency on VEGF expression in RPE and in turn on choroidal integrity are nearly identical to those observed in CD36-deficient animals (Figure 2).

## Discussion

Retinal degeneration and choroidal involution are cardinal features of the predominant, “dry” form of AMD. The molecular mechanisms that lead to these atrophic changes are not well known. In this study we show that CD36 deficiency causes photoreceptor/OS degeneration and choroidal involution in rats and mice. We show that CD36 expression is necessary for OS induced prosurvival/proangiogenic COX2 expression in RPE in vitro and that COX2 ablation causes similar choroidal involution in vivo. We propose a molecular mechanism that links photoreceptor degeneration and choroidal involution, the main features of dry AMD.

CD36 was expressed in mice in the basal aspect of the RPE and in choroidal vessels, as described in rat and human [11]. In contrast to data reported from in vitro experiments [12], CD36 does not seem to be essential for basal RPE phagocytosis in vivo, since an absolute defect in RPE phagocytosis would lead to a more rapid and complete retinal degeneration [8], whereas CD36 deficiency is associated with late-onset retinal degeneration. It has been suggested that CD36 plays a predominant role in OS phagocytosis mainly under oxidative conditions [13]. Interestingly, the relatively late morphological alterations observed in CD36-deficient animals seem to coincide with an increase in oxidative stress, as antioxidant defenses diminish with age [30]. This inference is reinforced by the accrued OS degeneration observed in oxidative stress-prone albino SHRs compared to pigmented CD36^−/−^ mice (Figure 1).

Choroidal involution was reported in SHRs several decades ago [27], prior to knowledge of their CD36 status. Our findings confirm the choroidal vascular rarefaction described in SHRs [27]. Furthermore, experiments using normotensive CD36-deficient animals [26] suggest that this rarefaction occurs independently of hypertension but seems secondary to CD36 deficiency. The deficient antiangiogenic signaling in the vascular endothelium due to the suppression of CD36 as the main receptor of TSP-1 [17] does not seem to significantly counterbalance this effect.

The normal appearance of capillary beds distant from the RPE, suggests a predominant role of local paracrine factors in CD36 dependent choroidal involution. In vivo, these CD36-dependent paracrine factors likely originate from the CD36-expressing RPE cells adjacent to the choriocapillaries. RPE cells express prosurvival/proangiogenic factors such as COX2 in vivo [23] that may be necessary for choriocapillary integrity. COX2 expression in the RPE is significantly augmented by retinal OS phagocytosis [15]. We hereby show that CD36 expression in RPE is necessary for the OS-induced expression of COX2 in RPE primary cultures, as CD36 deficiency blunted the OS response in vitro and diminished COX2 expression in vivo.

CD36 activation sufficed to induce COX2, suggesting that OS-induced COX2 expression in RPE cells is directly mediated by CD36 as recently described for oxidized low density lipoproteins (oxLDL) in COX2 expression in macrophages [31]. In macrophages, CD36 stimulation has been shown to activate the transcription factor nuclear factor kappa B (NF-κB) [32], which controls COX2 expression [33], and a similar mechanism might be involved in the RPE. Taken together, these results suggest that CD36 exerts an important permissive role in evoking the expression of prosurvival/proangiogenic factor COX2 in the RPE. We propose that choroidal involution is at least in part due to the observed COX2 down-regulation in RPE, since in our study COX2 deletion led to a similar choroidal involution. This inference is further substantiated by the interplay between COX2 and another major prosurvival/proangiogenic factor, VEGF, such that a COX2 activity deficiency (genetic and pharmacological) depressed VEGF immunoreactivity in RPE in vitro and in vivo, as seen in mice deficient in CD36, which itself also regulates both COX2 and VEGF expression. Together these findings suggest that diminished expression of CD36-dependent COX2 and VEGF in RPE might contribute to the rarefaction of the adjacent choriocapillaris.

All in all, our results show a novel molecular mechanism of photoreceptor degeneration and choroidal rarefaction, key cardinal features of dry AMD. Furthermore, our results suggest that pharmacological activation of CD36 or restoration of CD36 expression in the RPE of patients with dry AMD could be used therapeutically to prevent photoreceptor cell death by boosting SE renewal and to maintain a healthy choroid and retinal oxygenation by enhanced COX2 expression. These findings reveal a pathogenic process, to our knowledge previously unknown, with important implications for the development of new therapies for dry AMD.

## Abbreviations

AMD: age-related macular degeneration
ONL: outer nuclear layer
OS: outer segment
RPE: retinal pigment epithelium
RT: room temperature
RT-PCR: real-time PCR
SHR: spontaneous hypertensive rat
VEGF: vascular endothelial growth factor
